# Exaggerated Physiological Jaundice and Exchange Transfusion in Neonates

**DOI:** 10.7759/cureus.90083

**Published:** 2025-08-14

**Authors:** Muhammad Arif, Maria Himayat, Hina Nasir, Gul Ghutay, Shumaila Aziz, Huma Iftikhar, Saffaf Habib

**Affiliations:** 1 Pediatric Medicine, Northwest General Hospital and Research Centre, Peshawar, PAK

**Keywords:** exchange blood transfusion, hyperbilirubin, jaundice neonatal, newborn health, physiological jaundice

## Abstract

Background: Neonatal jaundice, particularly unconjugated hyperbilirubinemia, is a common condition that can lead to bilirubin-induced neurologic dysfunction (BIND) if severe. Physiological jaundice is typically benign, but exaggerated forms may necessitate interventions like phototherapy or exchange transfusion. This study aimed to determine the incidence and contributing factors of exaggerated physiological jaundice requiring exchange transfusion in neonates.

Methods: This retrospective case series analyzed medical records from January 2020 to January 2023 at Northwest General Hospital, Peshawar. Neonates with physiological hyperbilirubinemia (serum bilirubin ≥17 mg/dL) who underwent exchange transfusion were included. Data on demographics, clinical signs, feeding, and laboratory findings were extracted using a structured tool and analyzed with IBM SPSS Statistics for Windows, version 25.

Results: Out of 136 neonates with exaggerated physiological jaundice, 20 (14.7%) required exchange transfusion. The mean age at presentation was 5.2 ± 1.9 days. Males constituted 61.9%, and 81.0% were full-term. Exclusive breastfeeding was reported in 76.2% of cases. The mean total serum bilirubin at admission was 26.52 ± 7.7 mg/dL, reducing to 14.16 ± 2.6 mg/dL post-transfusion. Exclusive breastfeeding (76.2%), low birth weight (23.8%), and prematurity (14.3%) were identified as leading factors.

Conclusion: A significant proportion (14.7%) of neonates with exaggerated physiological jaundice required exchange transfusion. Exclusive breastfeeding, low birth weight, and prematurity were common contributing factors. These findings highlight the importance of early identification, close monitoring, and targeted management to reduce the need for aggressive interventions in this vulnerable population.

## Introduction

Jaundice is a clinical manifestation of hyperbilirubinemia, characterized by yellowish discoloration of the skin, sclera, and mucous membranes, typically observed when total serum bilirubin (TSB) levels exceed 5 mg/dL. It is one of the most common conditions encountered during the neonatal period. Approximately 60-70% of term and 80% of preterm neonates develop some degree of hyperbilirubinemia within the first week of life, with an estimated incidence of 8-11% requiring medical attention [1-5,6].

Hyperbilirubinemia may be conjugated or unconjugated. Conjugated hyperbilirubinemia is always considered pathological, whereas unconjugated hyperbilirubinemia may be either physiological or pathological [1]. Severe hyperbilirubinemia (TSB >25 mg/dL), regardless of etiology, poses a risk of bilirubin-induced neurologic dysfunction (BIND), including acute bilirubin encephalopathy. Clinical features include lethargy, poor feeding, vomiting, hypotonia, and seizures [1,6,7,8].

Neonatal jaundice is considered pathological when it presents within the first 24 hours of life, when TSB exceeds the 95th percentile for age in hours as per the hour-specific nomogram, when the bilirubin level rises faster than 0.2 mg/dL/hour or more than 5 mg/dL/day, when direct bilirubin exceeds 2 mg/dL, or when jaundice persists for more than two weeks in a term infant [1,9-10,11].

Common causes of pathological jaundice include immune-mediated hemolysis due to ABO or Rh incompatibility, increased red blood cell mass as seen in polycythemia, intracranial hemorrhage, infants of diabetic mothers, and impaired bilirubin clearance in conditions such as Gilbert syndrome types 1 and 2, Crigler-Najjar syndrome, and Lucey-Driscoll syndrome [10,11].

Physiological jaundice typically appears on the second day of life, peaks around days 3 to 5, and resolves by the second or third week [10]. The acceptable range of physiological bilirubin levels may vary due to multiple factors, including gestational age, birth weight, overall health status, hydration, nutrition, ethnicity, mode of feeding, and other epidemiological influences. Breastfeeding jaundice, often due to suboptimal feeding, presents during the first week of life. In contrast, breast milk jaundice, which occurs after the first week, is attributed to the transient immaturity of hepatic enzymes and is a common cause of prolonged physiological jaundice in exclusively breastfed neonates [8,11].

Physiological jaundice is generally a benign condition that rarely requires treatment. However, strict monitoring of serum bilirubin levels (SBR) is essential, as levels may rise to 17-18 mg/dL or higher [10,12]. Various studies have reported differing incidence rates of exaggerated physiological jaundice. One study found the incidence to be 56.3% without an identifiable cause [13], whereas another showed an incidence of 16.5% with a strong association with maternal infection [14]. As physiological jaundice is a diagnosis of exclusion, thorough clinical assessment is crucial. This includes a detailed maternal history, particularly of illnesses such as diabetes, and a complete physical examination of the newborn. Clinicians should look for evidence of bleeding (e.g., cephalohematoma, petechiae, or ecchymosis), signs of prematurity, polycythemia, and perform a neurological assessment for features of bilirubin-induced encephalopathy. Recommended laboratory investigations include total, direct, and indirect serum bilirubin levels; blood group and Rh typing of both mother and infant; the direct Coombs test; complete blood count; reticulocyte count; and serum albumin levels [15]. Management of exaggerated hyperbilirubinemia typically involves three approaches: phototherapy, pharmacological therapy, and exchange transfusion [15]. Phototherapy remains the most commonly used and effective method for reducing unconjugated hyperbilirubinemia, with approximately 62.6% of affected neonates requiring this treatment. Exchange transfusion is reserved for cases at high risk of neuronal toxicity [16]. A literature review indicated that 6% of neonates with unconjugated hyperbilirubinemia required exchange transfusion. Of these, 55.2% had pathological jaundice, commonly caused by ABO incompatibility, sepsis, or hypothyroidism, while 44.8% had exaggerated physiological jaundice, including 27.6% with no underlying pathology, 10.3% who were preterm, and 3.4% with cephalohematoma [17]. The main objectives of this study are to determine the current incidence of exaggerated physiological jaundice among neonates requiring exchange transfusion due to the risk of neuronal toxicity and to identify the most common contributing factors. These findings aim to guide improved management strategies and early interventions.

## Materials and methods

This retrospective case series was conducted in the neonatal nursery of Northwest General Hospital, Peshawar. The study included a review of medical records over a three-year period from January 2020 to January 2023. All neonates admitted to the nursery with a diagnosis of neonatal jaundice during this period were considered for inclusion. Neonates who had evidence of pathological hyperbilirubinemia were excluded from the study. Only neonates with physiological hyperbilirubinemia and a total serum bilirubin (SBR) level of 17 mg/dL or higher were included in the final analysis. Among these, cases that required exchange transfusion were further isolated for detailed evaluation. Data were extracted from hospital records using a structured data collection tool. The variables recorded included the neonate’s name, age at presentation, gender, birth weight, gestational age, mode of delivery, infant blood group, maternal blood group, type of feeding, and any antenatal maternal illness. Clinical signs were also evaluated, including evidence of infection in the neonate, signs of dehydration, manifestations of bilirubin-induced encephalopathy, and the presence of cephalhematoma, bruising, or hemorrhage. Laboratory investigations included total, conjugated, and unconjugated bilirubin levels on arrival; urea and electrolyte levels; complete blood count; serum albumin; C-reactive protein (CRP); and bilirubin levels following exchange transfusion.

The data were analyzed using IBM SPSS Statistics for Windows, version 25.0 (IBM Corp., Armonk, NY). Descriptive analysis was performed, and findings were presented using tables, graphs, and charts to summarize the results and highlight significant trends. The inclusion criteria were neonates who developed jaundice after the second day of life, had no laboratory evidence of hemolysis contributing to unconjugated hyperbilirubinemia, and underwent exchange transfusion for exaggerated physiological jaundice. Neonates were excluded if they were syndromic, developed jaundice within the first 24 hours of life, had laboratory evidence of hemolysis causing hyperbilirubinemia, or experienced jaundice persisting beyond the third week of life.

Ethical approval for the study was obtained from the Ethics Committee of Northwest General Hospital, Peshawar (approval no. 0119). Medical records were de-identified.

## Results

**Figure 1:**
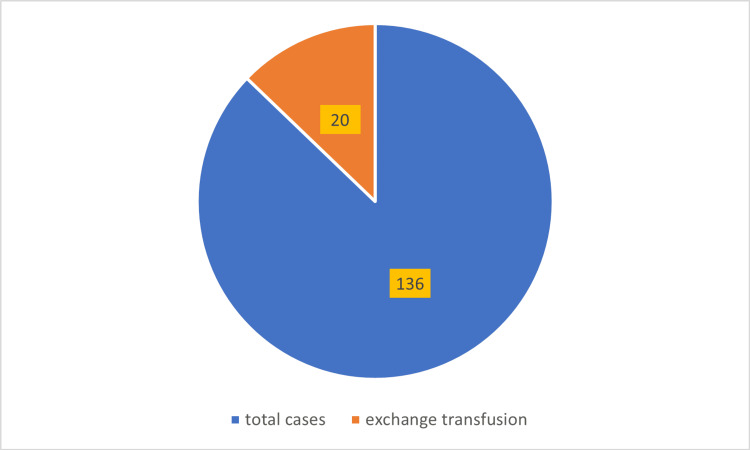
incidence of exchange transfusion in exaggerated physiologic jaundice

A total of 136 neonates were admitted with physiological jaundice and exaggerated unconjugated hyperbilirubinemia (serum bilirubin ≥17 mg/dL) over the three-year study period. Among these, 20 neonates required exchange transfusion, indicating an overall incidence of 20 (14.7%) for exchange transfusion in cases of exaggerated physiological jaundice, as shown in Figure 1.

Demographic and baseline clinical characteristics of the study population are presented in Table 1. Among the neonates who underwent exchange transfusion, the mean age at presentation was 5.2 ± 1.9 days (range 2-10 days). The mean birth weight was 2.96 ± 0.56 kg (range 1.70-3.90 kg). Low birth weight was seen in seven (33.3%) of cases, while 11 (52.4%) had normal birth weight, and two (9.5%) were macrosomic. The mean gestational age was 36.95 ± 2.04 weeks (range 32-38 weeks), with three (14.3%) being preterm and 17 (81.0%) full term.

**Table 1:** Demographic characteristics of neonates with exaggerated physiologic jaundice required exchange transfusion

Variables	Mean +- SD (min-max)	Frequency (percentages)
Age of baby on arrival (days)	5.2 ± 1.9 (2–10)	-
Weight of baby (kg)	2.96 ± 0.56 (1.70–3.90)	-
Low birth weight	-	7 (33.3%)
Normal birth weight	-	11 (52.4%)
High birth weight	-	2 (9.5%)
Gestational age (weeks)	36.95 ± 2.04 (32–38)	-
Preterm	-	3 (14.3%)
Full term	-	17 (81.0%)
Gender	-	-
Male	-	13 (61.9%)
Female	-	7 (33.3%)
Mode of delivery	-	-
Vaginal	-	16 (76.2%)
C-Section	-	4 (19.0%)
Feeding	-	-
Breastfeeding	-	16 (76.2%)
Breast plus bottle fed	-	3 (14.3%)
Exclusively bottle fed	-	1 (4.8%)

Of the neonates, 12 (61.9%) were male and seven (33.3%) were female. Vaginal delivery was the mode of birth in 16 (76.2%) cases, while four (19.0%) were delivered via cesarean section. Breastfeeding was reported in 16 (76.2%) neonates, while three (14.3%) received both breast and bottle feeding, and one (4.8%) was exclusively bottle-fed.

The distribution of blood groups among neonates and their mothers is shown in Table 2. Among neonates, the most common blood group was A positive (8; 38.1%), followed by O positive (6; 28.6%) and B positive (3; 14.3%). Maternal blood group distribution was similar, with A positive and B positive each seen in six (28.6%) mothers, followed by AB positive in five (23.8%).

**Table 2:** Blood groups of neonates

Baby blood groups	Number	Frequency (%)
A positive	8	38.1%
B positive	3	14.3%
AB positive	1	4.8%
O positive	6	28.6%
B negative	1	4.8%
O negative	1	4.8%

Laboratory findings for neonates who underwent exchange transfusion are summarized in Table 3. The mean total serum bilirubin at presentation was 26.52 ± 7.7 mg/dL, with indirect bilirubin averaging 25.27 ± 6.75 mg/dL and direct bilirubin 1.20 ± 0.98 mg/dL. Mean hemoglobin was 14.40 ± 4.24 g/dL, and hematocrit was 41.35 ± 11.5%. The mean C-reactive protein (CRP) was 1.49 ± 2.06 mg/L. Serum urea averaged 45.5 ± 14.84 mg/dL and sodium 142.0 ± 1.41 mmol/L. Post-exchange total serum bilirubin levels were reduced to a mean of 14.16 ± 2.6 mg/dL. The laboratory findings are presented in Table 4.

**Table 3:** Blood groups of mothers

Mother blood group	Number	Frequency (%)
A positive	6	28.6%
B positive	6	28.6%
AB positive	5	23.8%
O positive	2	9.5%
B negative	1	4.8%

**Table 4:** Laboratory findings

Findings (with units)	Mean ± standard deviation	Minimum – maximum
Total serum bilirubin (mg/dL)	26.52 ± 7.7	21.05 – 32.00
Indirect bilirubin (mg/dL)	25.27 ± 6.75	20.50 – 30.05
Direct bilirubin level (mg/dL)	1.20 ± 0.98	0.50 – 1.90
Hemoglobin (g/dL)	14.40 ± 4.24	11.40 – 17.40
Hematocrit (%)	41.35 ± 11.5	33.20 – 49.50
C-reactive protein (CRP) (mg/dL)	1.49 ± 2.06	0.03 – 2.95
Urea (mg/dL)	45.50 ± 14.84	35.00 – 56.00
Sodium (mmol/L)	142.00 ± 1.41	141 – 143
Total serum bilirubin after exchange transfusion (mg/dL)	14.16 ± 2.6	12.32 – 16.00

**Figure 2:**
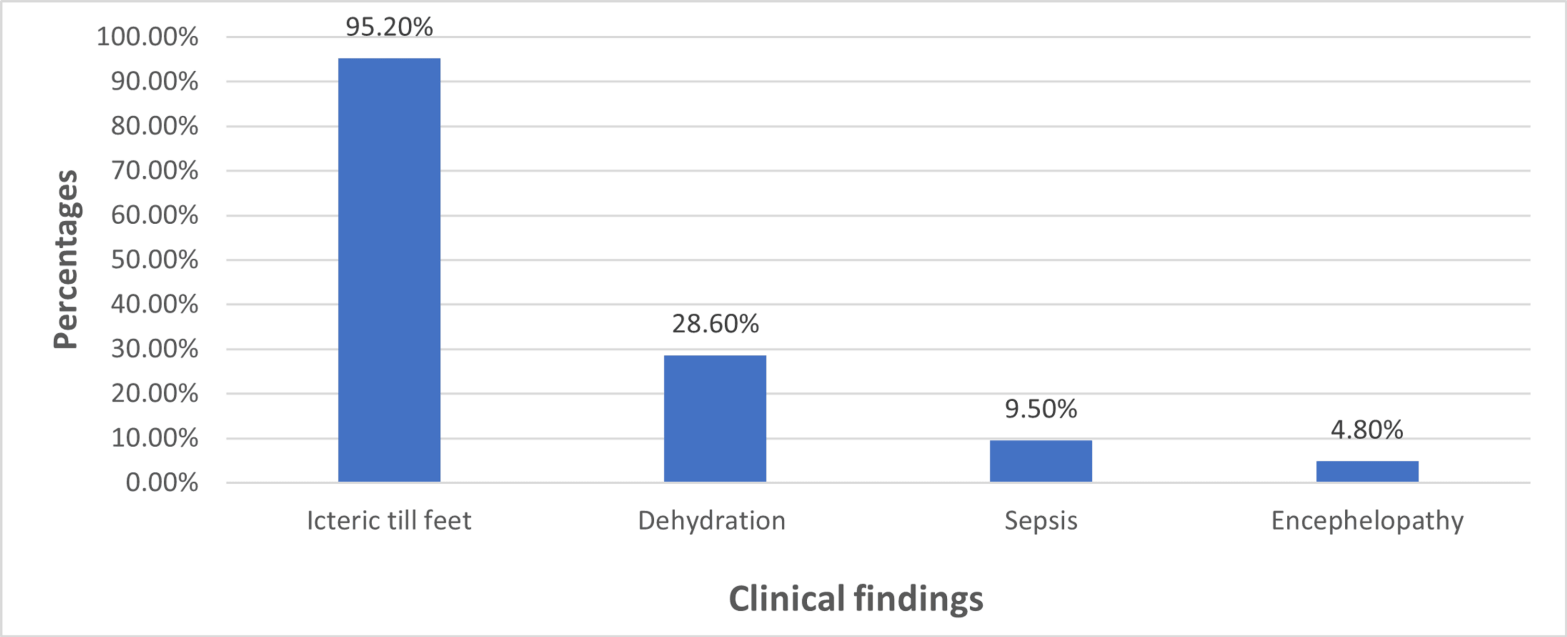
Percentages of various clinical findings

Figure 2 illustrates the percentage distribution of various clinical findings observed in neonates at the time of admission. The most common clinical sign was icteric till feet, followed by signs of dehydration and bruising. A smaller proportion of neonates exhibited features suggestive of bilirubin-induced encephalopathy.

**Figure 3:**
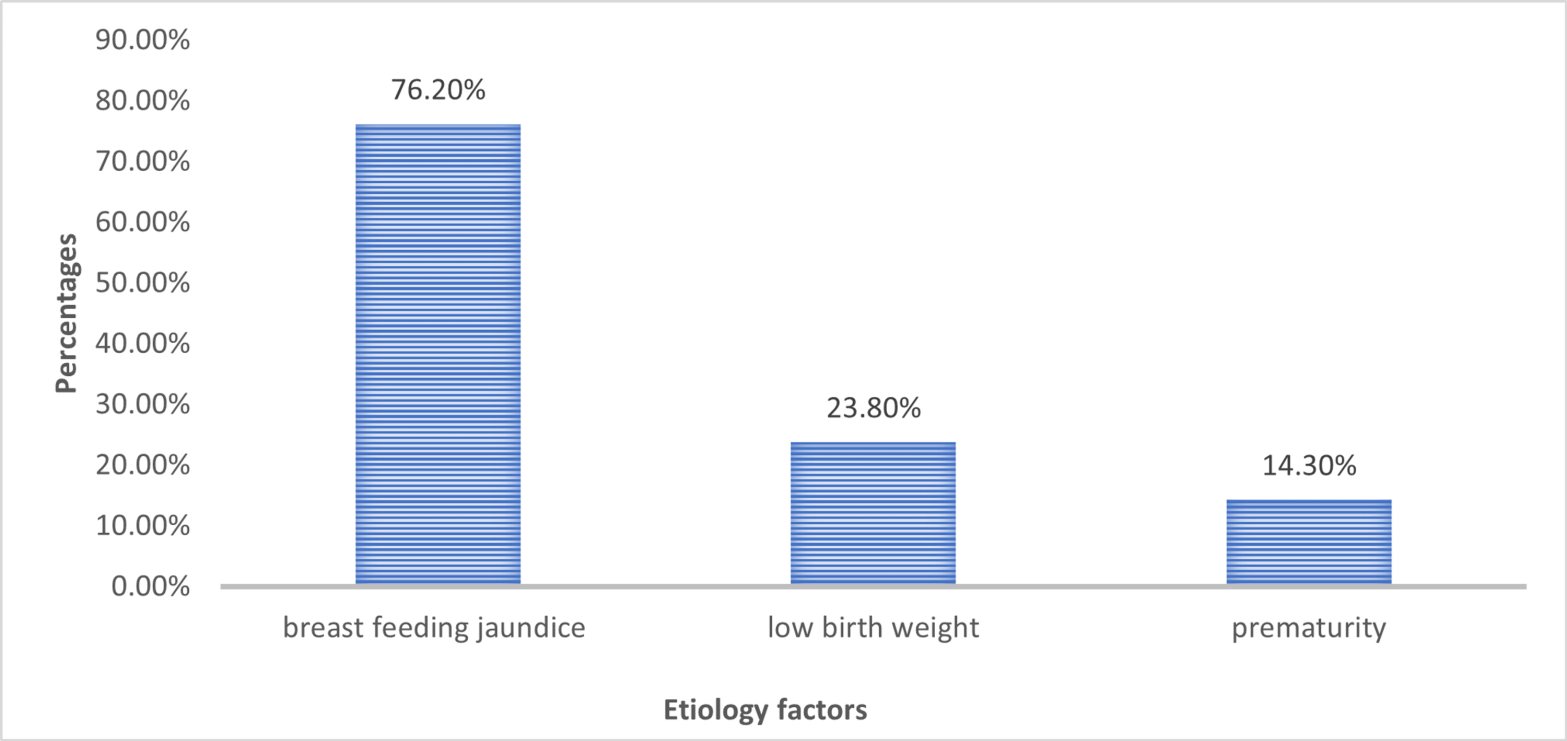
Frequencies of various etiology factors

Figure 3 presents the frequency of different etiological factors identified in cases of exaggerated physiological jaundice. In a significant number of neonates, no clear underlying cause was identified. Other contributing factors included prematurity, cephalhematoma, and maternal infection.

## Discussion

In this retrospective study conducted over a three-year period from January 2020 to January 2023 at Northwest General Hospital, a total of 136 neonates with exaggerated physiological jaundice were identified. Among these, 20 neonates (14.7%) required exchange transfusion in accordance with the American Academy of Pediatrics (AAP) guidelines for neonatal hyperbilirubinemia [18]. This incidence is notably lower than that reported in a study conducted in Nepal, which documented an incidence of 44.8% (19/42) for exchange transfusion among cases of exaggerated physiological jaundice, with an overall incidence of 6% (42/700) in all neonatal jaundice cases during a one-year period from March 2014 to March 2015 [19]. To date, there is limited literature specifically addressing the incidence of exchange transfusion in exaggerated physiological jaundice, underscoring the relevance of the current study.

In our study, the mean total serum bilirubin level at admission was 26.52 ± 7.7 mg/dL, which was reduced to a mean of 14.16 ± 2.6 mg/dL following exchange transfusion. The mean age at presentation was 5.2 ± 1.9 days, with the earliest presentation at two days and the latest at 10 days. Male neonates (61.9%) represented the majority of exchange transfusion cases, consistent with other studies that suggest a higher predisposition to jaundice in males [20]. Most of the neonates were full-term (81.3%), while 14.3% were preterm, with a mean gestational age of 36.95 ± 2.04 weeks. The mode of delivery also appeared to influence the incidence, with 76.2% (15/20) of affected neonates delivered vaginally and 19.0% (4/20) via cesarean section. Previous literature has similarly reported a higher incidence of physiological jaundice among neonates born vaginally, possibly due to increased bruising or birth trauma [21]. In addition, 76.2% (15/20) of neonates requiring exchange transfusion were exclusively breastfed. The condition was less common among neonates who were mixed-fed (14.3% or 3/20) or exclusively bottle-fed (4.8% or 1/20). These findings highlight the potential contribution of breastfeeding-associated jaundice and breast milk jaundice to exaggerated hyperbilirubinemia [22].

In terms of underlying etiologies, our study found that exclusive breastfeeding was the leading factor (76.2% or 15/20), followed by low birth weight (23.8% or 5/20) and prematurity (14.3% or 3/20). This aligns with existing data, including a previously reported study, in which 27.6% (15/54) of neonates requiring exchange transfusion had no identifiable cause and were presumed to have breast milk jaundice [19]. That same study also reported prematurity in 14.8% (8/54) and cephalhematoma in 3.4% (2/54) of cases. Interestingly, our study found no incidence of cephalhematoma among the exchange transfusion cases. Out of the 20 neonates who underwent exchange transfusion, one (4.8%) exhibited clinical signs of bilirubin encephalopathy, presenting with hypotonia and poor suck. A majority of neonates (96.2% or 19/20) were clinically icteric up to the soles, and 28.6% (6/20) showed signs of dehydration based on clinical examination and serum electrolyte values. In addition, 9.8% (2/20) of the cases had clinical and biochemical evidence of sepsis, indicated by elevated C-reactive protein levels. No antenatal maternal illness was identified in the cohort of neonates requiring exchange transfusion for exaggerated physiological jaundice.

These findings highlight the importance of early identification, close monitoring, and proactive management of neonates with physiological jaundice, especially those who are exclusively breastfed, of low birth weight, or preterm. Further prospective studies are recommended to better define risk factors and to standardize management protocols for exaggerated physiological jaundice to reduce the need for exchange transfusion [23,24].

The retrospective nature and small sample size are the limitations of our study. Multi-center, prospective studies with a large sample size will further explore this topic.

## Conclusions

In this retrospective analysis, newborns with accentuated physiological jaundice who were brought to the neonatal nursery over a three-year period had an overall frequency of 20 (14.7%) for exchange transfusion. The majority of these instances were full-term (110/136, 81.0%) and vaginally delivered (104/136, 76.2%), with a male predominance (84/136, 61.9%) and a mean age at presentation of 5.2 days. The most common contributing factor was exclusive breastfeeding (104/136, 76.2%), which was followed by prematurity (19/136, 14.3%) and low birth weight (32/136, 23.8%). These results emphasize important demographic and etiological correlations that guide better clinical surveillance and management options, as well as the noteworthy percentage of aggravated physiological jaundice patients that necessitate aggressive intervention.
